# Comparison of the Clinical Outcomes Between Isoperistaltic and Antiperistaltic Anastomoses After Laparoscopic Distal Gastrectomy for Patients With Gastric Cancer

**DOI:** 10.3389/fonc.2020.01237

**Published:** 2020-07-31

**Authors:** Yoontaek Lee, Chang Min Lee, Sungsoo Park, Jong-Han Kim, Seong-Heum Park

**Affiliations:** Department of Surgery, Korea University College of Medicine, Seoul, South Korea

**Keywords:** isoperistaltic, antiperistaltic, anastomosis, total laparoscopic, distal gastrectomy

## Abstract

**Background:** No consensus exists regarding the superiority of either of the two types of gastrointestinal anastomosis, which are isoperistaltic and antiperistaltic. This study aimed to compare the clinical outcomes between isoperistaltic and antiperistaltic anastomoses after total laparoscopic distal gastrectomy (TLDG) in patients with gastric cancer.

**Methods:** We retrospectively reviewed the medical records of patients with gastric cancer who underwent TLDG with Billroth II anastomosis between January 2014 and December 2018. The patients were divided into two groups according to the peristaltic direction of gastrointestinal anastomosis after TLDG. One group underwent isoperistaltic anastomosis (Iso group), and the other underwent antiperistaltic anastomosis (Anti group). Clinical outcomes were compared between the groups.

**Results:** Of the 148 patients who underwent TLDG with Billroth II anastomosis, 124 were included in the Iso group and 24 were included in the Anti group. The Anti and Iso groups showed no significant difference with regard to the incidence of internal hernia (0.0 vs. 6.5%, respectively; *p* = 0.355). The incidence of bile reflux was more frequent in the Iso group than in the Anti group (*p* = 0.010), but food stasis was more common in the Anti group than in the Iso group (*p* = 0.006).

**Conclusion:** In gastric cancer patients who underwent TLDG in which postoperative adhesion was minimized, antiperistaltic anastomosis may have created a physiologic barrier in gastrointestinal continuity. However, a large-scale study is necessary to validate the relationship between the digestive stream and the peristaltic direction.

## Introduction

Gastric cancer is still one of the most commonly diagnosed malignancies in East Asian countries. Evidence-based studies have shown that laparoscopic distal gastrectomy has better patient short-term outcomes and quality of life, with reduced pain and blood loss, earlier postoperative recovery, and shorter hospital stays, than open procedures (1). As a result, the current Japanese gastric cancer treatment guidelines have upgraded laparoscopic distal gastrectomy for clinical stage I cancer from an investigational treatment to an option in general practice (2). In addition, laparoscopic gastrectomy has been generally accepted as an alternative to open gastrectomy, and some experienced surgeons in specialized institutions have applied this technique in total gastrectomy or surgical treatment of patients with advanced gastric cancer.

Intestinal anastomoses were not routinely performed until Theodor Billroth demonstrated the feasibility and safety of intestinal anastomoses in the late 19th century (3). For anastomotic reconstruction after distal gastrectomy, Billroth-I, Billroth-II, or Roux-en-Y reconstruction is performed. In the Billroth-II or Roux-en-Y reconstruction method, isoperistaltic or antiperistaltic direction is selected according to the surgeon's preference. However, whether or not the direction of peristalsis has any influence over intestinal anastomosis in terms of postoperative complications and quality of life is unknown. Several articles favoring isoperistaltic anastomosis have affirmed that this method has advantages following operations on the esophagus and the hepatobiliary tract (4, 5). In contrast, some studies on gastrojejunostomy have reported fewer delayed gastric emptying cases after antiperistaltic reconstruction than after isoperistaltic reconstruction (6, 7). Despite the fact that there is no difference in the postoperative quality of life and nutritional status regarding bile reflux, it causes remnant gastric cancer resulting in mucosal inflammation and regeneration after Billroth-II reconstruction (8, 9). Therefore, evaluation of the functional effects of any of the peristaltic possibilities on gastrojejunostomy is important.

No studies have yet evaluated the functional effects of any of the peristaltic possibilities on gastrojejunostomy after distal gastrectomy in gastric cancer patients, and no consensus exists regarding whether one is superior to another in the two peristaltic possibilities, namely, isoperistaltic, and antiperistaltic. This retrospective study aimed to evaluate functional outcomes and complications according to the peristaltic direction after total laparoscopic distal gastrectomy (TLDG) followed by Billroth II reconstruction for gastric cancer.

## Materials and Methods

### Study Design and Patients

Between January 2014 and December 2018, 24 patients underwent TLDG with Billroth II gastrojejunostomy by antiperistaltic direction (Anti group) and 124 by isoperistaltic direction (Iso group) for primary gastric cancer in the Korea University Ansan Hospital, South Korea. The choice of peristaltic direction was determined by surgeon preference.

### Study Objectives

The primary objective of this study was bile reflux. The secondary outcomes were gastric food stasis, early postoperative complications, and internal hernia. Bile reflux and gastric food stasis in the remnant stomach were diagnosed by 1-year postoperative endoscopic findings after more than 9 h of *nil per os* (NPO). Endoscopic findings were retrospectively reviewed by an experienced gastroenterologist who evaluated patients for bile reflux and gastric residue in the remnant stomach using a scoring system [Residue, gastritis, Bile (RGB) classification] reported by Kubo et al. (10). We defined bile reflux as “when a yellow liquid was observed in the remaining stomach” and gastric food stasis as grade 2 or higher according to RGB classification.

### Surgical Procedures

TLDG procedures were performed in all cases. A patient was placed under general anesthesia with legs separated. The operator sat on the right side of the patient, and the first assistant was positioned on the left side. The scope assistant was positioned between the patient's legs. A 12-mm trocar was inserted through a transumbilical incision using an open method. A flexible scope was inserted through this umbilical port after a pneumoperitoneum was created. Under the guidance of the flexible scope, a 5-mm trocar was placed at the right subcostal margin, and a 12-mm trocar was placed at the right midclavicular line. The first assistant inserted two 5-mm trocars at the left subcostal margin and left midclavicular line. The intraabdominal pressure was maintained at a constant 15 mmHg.

**Figure 1:**
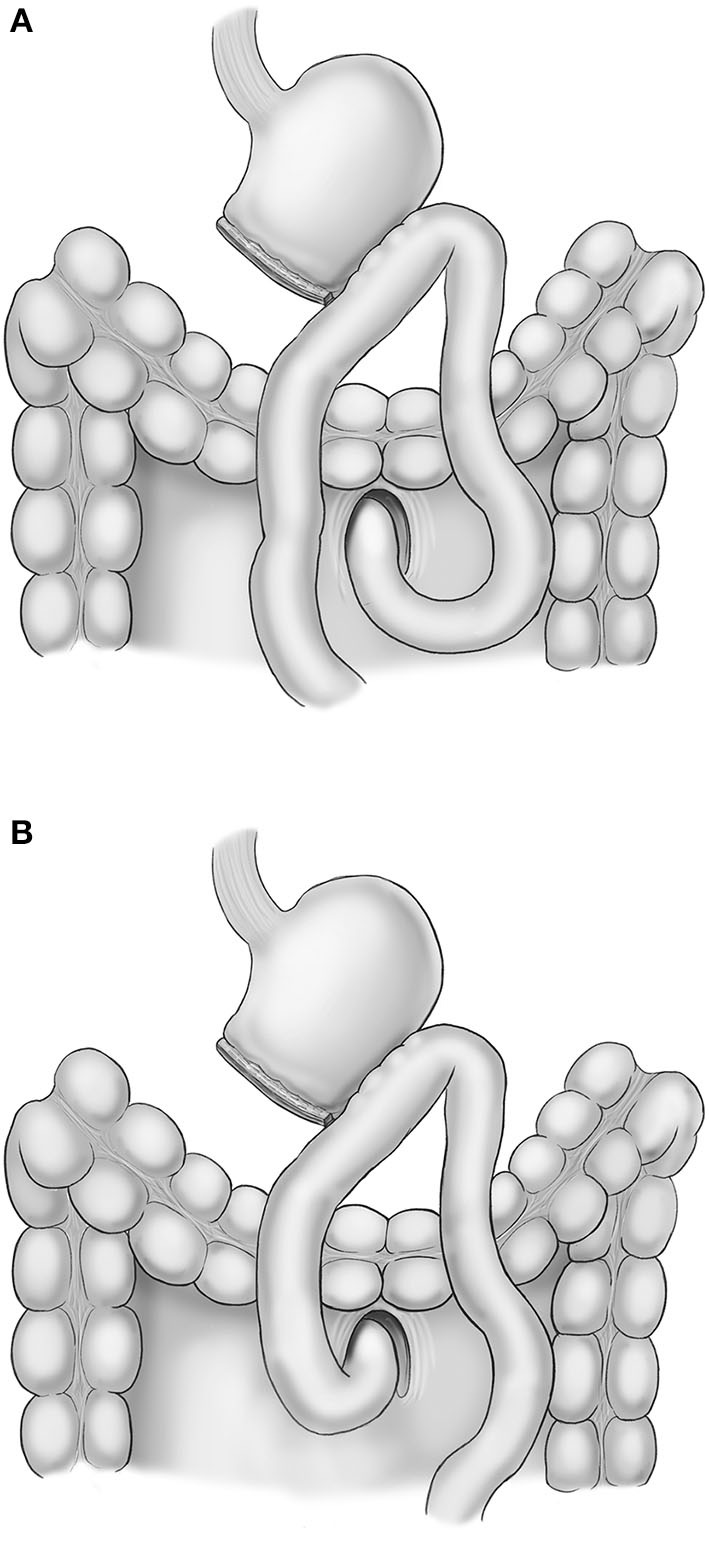
Scheme of isoperistaltic gastrojejunostomy **(A)** and antiperistaltic gastrojejunostomy **(B)**.

Lymphadenectomy for curative distal gastrectomy was accomplished based on the criteria of the Japanese Gastric Cancer Treatment Guidelines 2014 (ver. 4) (2). After lymphadenectomy completion, Billroth II gastrojejunostomy was performed for recovery of gastrointestinal continuity. All reconstructions were performed in antecolic fashion, and the peristaltic direction was based on the surgeon's preference (Figure 1). Braun jejunojejunostomy was also performed to reduce bile reflux into the remnant stomach. All anastomoses were performed with laparoscopic linear staplers (Endo GIA® Medtronic, Minneapolis, MN, USA). The common entry hole was closed by a laparoscopic suture technique using barbed thread.

The mesenteric defect of jejunojejunostomy and Petersen defect were not routinely closed in isoperistaltic anastomosis until August 2016. However, all mesenteric defects have been closed using non-absorbable suture in isoperistaltic anastomosis since September 2016. All mesenteric defects were not routinely closed in antiperistaltic anastomosis based on the surgeon's preference.

### Data Collection

Patient data were collected from electronic medical records. Clinicopathologic features, including age, sex, body mass index (BMI), American Society of Anesthesiologists (ASA) score, operative time, time to first diet, tumor depth, number of retrieved lymph nodes, and postoperative complications, were investigated. Postoperative complications, including wound infection, leakage, and intestinal obstruction occurring within 30 days of surgery, were evaluated according to the Clavien–Dindo classification (11).

### Statistical Analysis

Continuous variables are presented as mean ± standard deviation or median (interquartile range). Statistical analyses were performed using the chi-square test for the categorical variables and the Mann–Whitney *U*-test for the continuous variables. A *p*-value threshold of 0.05 was considered statistically significant. All statistical analyses were performed with R software (R Foundation for Statistical Computing, Vienna, Austria; http://cran.r-project.org/).

### Ethics Statement

The institutional review board of the Korea University Medical Center Ansan Hospital (2018AS0270) approved the present study, and the need for individual informed consent was waived by the ethics committee because of the use of anonymized data. All of the procedures were in accordance with the ethical standards of the responsible committees on human experimentation (institutional and national) and with the 1964 Declaration of Helsinki and later versions.

## Results

### Patient Characteristics

Of the 148 patients who underwent TLDG with Billroth II gastrojejunostomy, 24 were included in the Anti group and 124 were included in the Iso group. Table 1 shows the baseline characteristics of the patients. Age, sex, ASA score, and TNM stage were not significantly different between the two groups. The BMIs were 22.5 and 24.1 kg/m^2^ in the Anti and Iso groups, respectively (*p* = 0.024).

**Table 1:** Clinicopathogical characteristics of patients.

	**Anti (*n =* 24)**	**Iso (*n =* 124)**	***P*-value**
Age (yr)	59.1 (12.8)	61.9 (11.9)	0.311
Sex			0.632
Female	6 (25.0)	39 (31.5)	
Male	18 (75.0)	85 (68.5)	
Body mass index (kg/m^2^)	22.5 (3.7)	24.1 (3.0)	0.024
ASA performance status			0.441
1	7 (29.2)	21 (16.9)	
2	14 (58.3)	87 (70.2)	
3	3 (12.5)	15 (12.1)	
4	0 (0.0)	1 (0.8)	
pStage.			0.194
I	13 (54.2)	90 (72.6)	
II	4 (16.7)	13 (10.5)	
III	7 (29.2)	21 (16.9)	

*Data shown are number (%), mean (SD), or median (IQR)*.

*Anti, anti-peristatic; Iso, iso-peristatic; ASA, American Society of Anesthesiologists; SD, standard deviation; IQR, interquartile range*.

### Surgical Outcomes

Table 2 presents the postoperative surgical outcomes. The mean operation times were 251.9 and 232.3 min in the Anti and Iso groups, respectively (*p* = 0.055). The mean numbers of harvested lymph nodes were 51.0 and 44.0 in the Anti and Iso groups, respectively (*p* = 0.192). The time to the first diet in the Anti group was significantly longer than that in the Iso group [6.0 (5.5–7.5) vs. 5.0 (5.0–6.0) days, respectively, *p* < 0.001]. Postoperative complications of grade III or more occurred in four (16.7%) patients in the Anti group and one (0.8%) in the Iso group (*p* = 0.002).

Internal hernia was detected in eight patients in the Iso group but no patients in the Anti group. However, the incidence of internal hernia showed no significant difference between the Anti and Iso groups (0.0 vs. 6.5%, respectively; *p* = 0.355). Table 3 shows the characteristics of the eight patients with internal hernia in the Iso group. The median time to internal hernia detection was 20 months (3–48 months). Two of these eight patients were diagnosed with internal hernia on routine CT scan; these two were asymptomatic and had not undergone reoperation. In the other six patients, the hernia orifice was the jejunojejunostomy defect in one patient and the Petersen defect in five patients. Four patients underwent laparoscopic reduction of the hernia and closure of the defects; however, the remaining two required small bowel resection for ischemia.

**Table 2:** Surgical outcomes.

	**Anti (*n =* 24)**	**Iso (*n =* 124)**	***P*-value**
Operation time (min)	251.9 (42.9)	232.3 (46.0)	0.055
Number of harvested LNs	51.0 (24.5)	44.0 (18.1)	0.192
Time to the first diet (day)	6.0 (5.5–7.5)	5.0 (5.0–6.0)	<0.001
Complications within 30 days (grade III or more)^*^: yes	4 (16.7)	1 (0.8)	0.002
Intraabdominal abscess	1	1	
Anastomosis leakage	1	0	
Duodenal stump leakage	1	0	
Pleural effusion	1	0	
Internal hernia	0 (0)	8 (6.5)	0.355

*Data shown are number (%), mean (SD), or median (IQR)*.

*Anti, anti-peristatic; Iso, iso-peristatic; LNs, lymph nodes; SD, standard deviation; IQR, interquartile range*.

* *According to the Clavien–Dindo grading system*.

**Table 3:** Characteristics of patients who underwent internal hernia.

**Sex**	**Age**	**Stage**	**Time to detection of internal hernia(month)**	**Hernia orifice**	**Closure of Hernia orifice during primary gastrectomy**	**Reoperation**	**Full recovery**
M	68	I	6	Jejunojejunostomy	No	Laparoscopic reduction	Yes
M	74	II	4	Petersen	No	Laparoscopic reduction	Yes
M	59	I	26	Petersen	No	Laparoscopic reduction	Yes
M	65	I	20	Petersen	No	Open small bowel resection	Yes
M	50	I	48	Petersen	No	Open small bowel resection	Yes
M	53	I	3	Not identified	No	Not performed	Yes
M	63	I	25	Petersen	Yes	Laparoscopic reduction	Yes
M	60	II	10	Not identified	Yes	Not performed	Yes

### Endoscopic Evaluation at Postoperative 1 Year

The incidence of bile reflux in the Anti group was significantly less than that in the Iso group [3 (12.5%) vs. 51 (41.1%), respectively; *p* = 0.010] (Table 4). However, residual food was more frequently observed in the Anti group than in the Iso group [5 (20.8%) vs. 4 (3.2%), respectively; *p* = 0.006].

**Table 4:** Endoscopic evaluations of bile reflux and residual food.

**Postoperative 1year**	**Anti (*n =* 24)**	**Iso (*n =* 124)**	***P*-value**
Bile reflux	3 (12.5)	51 (41.1)	0.010
Residual food	5 (20.8)	4 (3.2)	0.006

*Data shown are number (%)*.

*Anti, anti-peristatic; Iso, iso-peristatic; SD, standard deviation; IQR, interquartile range*.

## Discussion

This study aimed to evaluate functional effects, including bile reflux and gastric food stasis, according to the peristaltic directions on gastrojejunostomy after distal gastrectomy in gastric cancer patients. Compared to antiperistaltic anastomosis, isoperistaltic anastomosis is a more natural method of restoring intestinal continuity; several studies have affirmed the advantages of isoperistaltic anastomosis on the esophagus and the hepatobiliary tract (4, 5). However, these studies are not applicable in the case of gastrojejunostomy or ileocolic anastomosis because antiperistalsis anastomosis of colonic esophageal reposition or hepaticojejunostomy causes gastrocolic reflux or ascending cholangitis due to peristalsis in the opposite direction of food or bile. In contrast, in the case of gastrojejunostomy or ileocolic anastomosis, the food passage direction is the same, but the synchronization of the peristalsis between the proximal and distal parts of the anastomosis may differ.

Regarding the direction of peristalsis for gastrojejunostomy, the first report on gastrojejunostomy for palliation of gastric outlet obstruction showed fewer cases of gastric food stasis after antiperistaltic reconstruction than after isoperistaltic reconstruction (6). A study on Roux-en-Y gastrojejunostomy after distal gastrectomy showed that antiperistaltic reconstruction is associated with a reduction in delayed gastric emptying (7). The reason for gastric food stasis reduction with antiperistaltic reconstruction is the flow direction of gastrojejunostomy. A Japanese study showed on contrast radiography that a straight flow direction of gastrojejunostomy is important in reducing gastric food stasis (12). This study presented that antiperistaltic anastomosis tends to be associated with a straight flow direction of gastrojejunostomy, reducing gastric food stasis. Similarly, a randomized controlled trial for ileocolic anastomosis after right hemicolectomy showed that patients with antiperistaltic anastomosis have a shorter intestinal transit time compared to those with isoperistaltic anastomosis (13).

However, the present study showed that antiperistaltic reconstruction is significantly associated with delayed gastric emptying and bile reflux reduction. A possible explanation for this mismatch with the findings of previous studies is the difference in the reconstruction method. The afferent and efferent loops are anchored to the anastomosis site in the Billroth II method; hence, the flow direction in the efferent loop is different from that in the Roux-en-Y method. Focusing on bile reflux, a possible explanation for the bile reflux reduction with antiperistaltic reconstruction is the mechanism of a physiologic barrier in gastrointestinal continuity. Food stasis arising from antiperistaltic anastomosis acts as a barrier to bile reflux. This concept is consistent with the theory of “functional pseudovalvular mechanism for antiperistaltic and colonic anastomosis.” The antiperistaltic direction in ileocolic anastomosis may act like a functional pseudovalve reducing ileocecal reflux and postoperative ileus (13).

The present study has some limitations. First, an inherent selection bias exists with regard to the surgical procedure because of the retrospective nature. Second, this study was performed by two surgeons from a single institution. Therefore, technical factors may have affected surgical outcomes. Third, the Anti group was composed of a relatively small number of patients; thus, a multicenter study with a larger population of patients is warranted to more effectively evaluate the functional outcomes according to the peristaltic direction.

In conclusion, in gastric cancer patients who underwent TLDG in which postoperative adhesion is minimized, antiperistaltic anastomosis may reduce bile reflux, creating a physiologic barrier in gastrointestinal continuity. However, a larger scale study is necessary to demonstrate the relationship between the digestive stream and the peristaltic direction.

## Data Availability Statement

The raw data supporting the conclusions of this article will be made available by the authors, without undue reservation.

## Ethics Statement

The studies involving human participants were reviewed and approved by The Institutional Review Board of the Korea University Medical Center Ansan Hospital (2018AS0270) approved the present study and the need for individual informed consent was waived by the ethics committee. Written informed consent for participation was not required for this study in accordance with the national legislation and the institutional requirements.

## Author Contributions

CL designed the study. YL conducted the research in combination with analyses of the data. YL and CL wrote the main manuscript text. SP, J-HK, and S-HP edited the manuscript. All authors read and approved the final manuscript.

## Conflict of Interest

The authors declare that the research was conducted in the absence of any commercial or financial relationships that could be construed as a potential conflict of interest.
